# Hidden in plain sight: antisemitic content in QAnon subreddits

**DOI:** 10.1371/journal.pone.0318988

**Published:** 2025-03-19

**Authors:** Dana B. Weinberg, Meyer D. Levy, April Edwards, Jeffrey S. Kopstein, David Frey, Peter Antonaros, Nikola Baci, Noah D. Cohen, Javier A. Fernandez, Yunis Ni

**Affiliations:** 1 Department of Sociology, Queens College-CUNY, Queens, New York, United States of America; 2 Department of Cyber Science, United States Naval Academy, Annapolis, Maryland, United States of America; 3 Department of Political Science, University of California Irvine, Irvine, California, United States of America; 4 Center for Holocaust and Genocide Studies, United States Military Academy at West Point, West Point, New York, United States of America; 5 Department of Applied Physics and Applied Mathematics, Columbia University, New York, New York, United States of America; 6 Department of Criminal Justice, John Jay College-CUNY, New York, New York, United States of America; 7 Department of Sociology, Princeton University, Princeton, New Jersey, United States of America; Tokyo Institute of Technology: Tokyo Kogyo Daigaku, JAPAN

## Abstract

When is online content antisemitic? This matter is highly contested, except in the case of explicit language. Yet implicit antisemitic content and conspiracy narratives about Jews have been on the rise, especially on moderated platforms. This paper maps empirically the connections between explicit antisemitic content and these other forms of content, showing the language game at play in an online community identified as antisemitic and providing a relatively simple answer to the classification of content question. Using data from two QAnon subreddits, r/CBTS_Stream and r/greatawakening, we identify the co-occurrence of explicit and implicit antisemitic language posted to the subreddits. The language game involves an ingroup having specialized knowledge related to implicit language or dog whistles; the ingroup knows and uses the secret meaning of these terms as an insider’s code. Content network analysis and qualitative coding illustrate that QAnon taught this insider’s code by presenting the overt, antisemitic meanings of implicit terms and generalized narratives in posts that combined them with explicit language. While explicit language appeared rarely and was used by only a small proportion of users, more than a third of QAnon users employed implicit antisemitic language in their posts. This implicit language communicated antisemitic conspiracy narratives and antisemitic ideas more generally, to an audience “in the know” while also offering the user plausible deniability. Moreover, the implicit antisemitic terms circumvent platform censorship and provide an opportunity to leverage common ground around antisemitic conspiracy narratives with new users without the stigma of explicitly antisemitic content. The question of whether content is antisemitic may easily be answered by looking at a community’s posts that combine explicit and implicit antisemitic language. (272 words).

## Introduction

When is online content antisemitic? This is a hotly contested issue but an increasingly important one as violence against Jewish communities has been rising alongside content purported by different groups to be antisemitic [1,2]. Some of this content is explicit or overt in its expression of hatred and calls for violence while other examples rely on veiled or implicit references. Herein we provide the first study analyzing the relationship between explicit and implicit language (otherwise known as "dog whistles") within an antisemitic language community. Methodologically, we provide a new way of studying this language game that is portable to other cases. In doing so, we highlight the important role conspiracy theory narratives play in solidifying a linguistic community and facilitating the spread of on-line hate.

In this paper, we use the case of antisemitic content to offer a community-based methodology for understanding the intention behind implicit references, one that is also applicable to other cases of hate. We argue that both explicit and implicit antisemitic content relate to one another through the language games communities play as they construct their ingroup identity in relation to an antagonistic outgroup. We show empirically both the differences in the way these types of content are deployed and the connections between them—both in terms of co-occurrence and in terms of signaling well-known conspiracy narratives—that facilitate widespread dissemination of hate.

Commentators and scholars have drawn the connection between on-line antisemitic conspiracy narratives and off-line violence [3]. Researchers have found that the proportion of antisemitic content greatly increases after major political events, such as the 2016 U.S. Presidential election, and acts of hate, such as the “Unite the Right” rally in Charlottesville, Virginia  in 2017, and other events referenced in the news [4–6]. Further connection can be inferred from the escalating dynamic between hate speech and violence, such as the massacre at the Pittsburgh Tree of Life Synagogue on October 27, 2018, the most violent antisemitic attack in US history, which was carried out by a perpetrator who had used a social media platform to spread antisemitic conspiracy theories in the run up to the attack. These events coincide with increased concerns among Jews about antisemitism in the US and attendant anxiety about violence [7]. The outpouring of online antisemitism in the months after the Hamas attack on Israel on October 7, 2023, shocked much of the American establishment and opened the possibility of a shift from on-line hatred to off-line violence, as the ADL reported nearly a 340% increase in antisemitic incidents in the two-months following the October 7^th^ attack [8].

Although there is little agreement on what constitutes antisemitic content, research conducted by policy groups points to the pervasiveness of antisemitic discourse in online platforms [9]. Mulhall (2021) found, for example, that in Europe every social media platform contained a wide range of easy-to-find antisemitic content. Greater quantities of explicit and overt antisemitism could be found on platforms with laxer policies and lighter moderation [9]. Twitter (now X) saw an explosion of antisemitic content after Elon Musk eased platform content moderation policies. Even with stronger oversight, content moderation by platforms is limited in detection capabilities [10] and does not seem to stop antisemitic posts and discussion. Instead, users adapt their discursive practices to avoid platform detection. Rather than use overtly antisemitic language, they turn to antisemitic conspiracy tropes [9]:

Jew-hatred has such deep roots within conspiracy ideologies that antisemitic tropes are rarely far removed from a diverse array of conspiratorial notions.... Indeed, such tropes pervade the genre to the extent that, for some, the role of the supposed Jewish conspirators is implicitly understood and does not need to be identified by name. Conspiracy ideologies are malleable, and adherents can choose to minimise certain aspects, such as antisemitism, in order to maximise its appeal to the uninitiated. Many individuals may ignorantly regurgitate antisemitic tropes unaware that they are racist, or turn a blind eye and deny such charges as a smear. [p. 15]

Conspiratorial tropes, thus, provide an implicit rather than overt or explicit way to invoke antisemitic messages. Moreover, feelings of political powerlessness, upon which conspiracy theories often rely [11], are strongly connected to feelings of directed antipathy towards Jews [12,13]. As a result, users familiar with antisemitic tropes may quickly recognize Jews, whether mentioned indirectly or not at all, as the key conspirators. Such implicit content neatly avoids any violation of platform rules or policies, and users have plausible deniability that they are promoting anti-Jewish hate.

In contrast to the policy group reports described above, peer-reviewed research seeking to track the pervasiveness and spread of antisemitism has focused almost exclusively on explicit content. Keywords play an important role in hate speech detection [14], and researchers have sought to identify terms and rhetoric that signal antisemitic content [5,14,15] using a variety of computational approaches. These efforts reflect the findings in the policy reports about the increase in antisemitic content online, and they have also yielded an expanded lexicon of slang and ethnic and racial slurs beyond immediately obvious terms. Chandra et al. note that their approach underperformed in cases of expressions of subtle hate, or what we would term implicit content, as well as sarcasm and trolling. Moreover, though Zannettou et al. identify explicit terms that reference biblically-based antisemitic conspiracy theories [5] and Ali and Zannettou identify explicit terms related to Holocaust denial [15], neither study unearths either the extensive use of more modern conspiracy theories or the use of more subtle language referenced in the policy reports described above. Together, these peer-reviewed studies capture an expanded net of explicitly antisemitic content, including images, but they have been less successful in identifying implicit terms or rhetoric as antisemitic. This paper contributes to knowledge about the online presentation and spread of antisemitism as well as other types of hate by specifically addressing the role of implicit content.

Using implicit rhetoric allows conspiracy narratives to continue to spread and gain audience, including among those who might initially find overt hate content unpalatable. Moreover, the internet has contributed to the growing acceptance of conspiracy theories, moving them from the fringe to the mainstream [16] and thereby granting them greater legitimacy and increasing the possibilities for violence [17]. As users engage with these conspiracy narratives, however, they are primed to follow the narrative network to increasingly overt content where the conspirators are explicitly identified as Jews. As Sutton and Douglas [18] describe, many conspiracy theory believers appear to experience what the authors call “Rabbit Hole Syndrome,” or quick acceleration in the discovery and assimilation of available conspiracy theory thinking. Such unfolding often involves the taking in of antisemitic beliefs in particular. For example, studies of YouTube channels belonging to conspiracy theorists David Icke, Ken O’Keefe, and Richie Allen found that while antisemitic content was not explicitly part of their videos, audiences often brought such opinions with them to comment sections [19,20]. Similarly, Garner, McGrann, Klug, et al. [21] found that the COVID-19 conversation on Twitter was strongly intertwined with antisemitic conspiracy tropes. These studies show that when users explore conspiracy theories of interest, explicitly antisemitic blame is likely to be close at hand. Weinberg and Dawson argue that connections between such themes form a narrative network which enables the smooth transition from one conspiracy theory to another [22]. This dynamic is potentially very dangerous, as the introduction of anti-minority language, even mildly, at first, can lead to escalation, dehumanization, and desensitization that can turn rhetoric into open intergroup contempt and to discriminatory views and norms [23].

What is the relationship between explicit antisemitic content and these more subtle forms presented through implicit language or conspiracy narratives? The research reviewed here assumes that implicit language and narratives are used cunningly as substitutes for explicit language to avoid platform detection. But how do users know to use these other forms of expression when they are not easily recognized by computational models, and how do others decode them to read the antisemitic messages they contain if there is plausible deniability? In what follows, we explore how antisemitic meanings of implicit terms and conspiracy narratives are established in an online community. We formalize a description of the relationships between explicit language, implicit language, and conspiracy narratives as hypotheses that we then test using two QAnon subreddits as our test bed. In so doing, we elaborate empirically the mechanisms through which antisemitic hate and prejudice are communicated and spread in online communities.

## Antisemitic attitudes, conspiracy narratives, and the language game

What is the theoretical connection between explicitly antisemitic attitudes and expression, antisemitic conspiracy narratives, and implicit language that does not specifically mention Jews? We begin by describing the relationship between dimensions of antisemitism commonly measured as attitudinal items on surveys, antisemitic conspiracy narratives that reflect these dimensions, and community-based language games. These language games frame Jews as a dangerous outgroup while utilizing implicit language or “dog whistles” to communicate this information to the ingroup and keeping others, including platform moderators, in the dark. We then offer a series of hypotheses to be tested in the paper.

### Four Dimensions of Antisemitic Attitudes

In addition to its status as “the oldest hatred,” antisemitism is unique among modern racial, ethnic, and religious antipathies [24]. It is a multidimensional concept that scholars have grouped into four broad dimensions, each of which may filter its way into popular discourse [25,26]. Each of these dimensions offer “reasons” for antipathy toward Jews. Each has a long pedigree, in some cases decades or even centuries. Furthermore, each has elements that appeal potentially to the political right [27] and left [28,29], among Christians and Muslims [30], and racial majorities and minorities [31]. For this reason, scholars have repeatedly maintained that antisemitism is one of the few hatreds that crosses the otherwise highly polarized partisan divide in the US and elsewhere [32–34]. Survey researchers have measured each of these dimensions for decades with a battery of well-tested questions. These dimensions of antisemitic attitudes refer to: hidden Jewish power, dual loyalty, Holocaust minimization or obfuscation, and distasteful Jewish traits and behaviors. We describe each below.

#### Hidden Jewish power.

Jews are frequently depicted in antisemitic discourse as the ones pulling the strings behind the scenes. Their hidden power may be political (in parties or as funders of parties), economic (Jewish bankers, finance, captains of industry), or cultural (Jewish journalists, academics, New York intellectuals, social media moguls, and Hollywood personalities). These attitudes are captured in surveys using such basic prompts as “Jews have too much power in finance” or “Jews have too much control over the U.S. Government” [35].

#### Dual Loyalty.

Antisemitic discourse often portrays Jews as insular and/or not caring about anyone but their own kind. This dimension questions the loyalty of Jewish citizens to their own communities, to their country, and frequently focuses on the Jewish connection to the state of Israel. The attitudes are gauged by such prompts as “Jews don’t care about anyone but their own kind” or “Jews are more loyal to Israel than to the United States” [36].

#### Holocaust Minimization or Obfuscation.

Antisemites are prone in their public discussion to question or deny that six million Jews died during the Holocaust or that it even occurred at all. On the one hand, Holocaust denial or minimization is a way of whitewashing Nazis and right-wing extremists; on the other hand, it is a way of depicting supposedly already rich and powerful Jews as using the sympathy and compassion of non-Jews for their own nefarious ends, whether for reparations, support for communal institutions, or aid for Israel. Survey researchers tap into these attitudes with the prompts “Jews use the Holocaust for advantage in international politics” or “Jews talk too much about the Holocaust” [35,37]. This minimization and obfuscation have been compounded by the rise of pseudo-scientific “information” utilized to refute or argue Holocaust denial as a legitimate framework [38].

#### Distasteful Jewish traits and behaviors.

In some ways, this dimension of antisemitic discourse is the oldest and most capacious. In antiquity and the medieval world Jews were charged with misanthropy, deicide (the murder of Jesus), host desecration and the use of Christian blood for ritual purposes (the “blood libel” and also “satanic rituals”), and with undue valuing of the literal and material over the spiritual and eternal [39]. In modern times, some of these traditional prejudices remain, but others have been translated into distasteful images of Jews as greedy, pushy, and devious swindlers. Survey researchers measure distribution of these attitudes with prompts such as “Jews can’t be trusted in business” and “Jews use the blood of Christians for ritual purposes.” [40,41].

At the extreme, these four dimensions dehumanize or demonize Jews and thus make them ready villains—wielding the secret hand of power, betraying others for their own interests, manipulating others through (false) victimhood, and/or sacrificing children—for sinister and dangerous conspiracies on a global level. Moreover, following Intergroup Threat Theory, these dimensions may be used to characterize Jews as an outgroup that poses both realistic threats (related to economic or material, physical, or political resource competition) and symbolic threats (related to differences in values, norms, and beliefs) to the ingroup [42], providing a basis for prejudice and a desire to protect the ingroup from this perceived outgroup threat [43]. Finally, even when unacceptable stereotypes associated with these dimensions are discredited, their invocation may cue other stereotypes and consequently contribute, though indirectly, to discrimination [44].

### Antisemitic conspiracy narratives

Each of the four dimensions of antisemitism can be measured and tallied in relation to attitudes, and scholars mostly do so with additive indices. This approach makes eminent sense as a quick and efficient way of gauging the extent and distribution of individual antisemitic attitudes, but it does not adequately capture how antisemitism is expressed in everyday communication. Nor does it capture how antisemitism spreads, that is, how it “works.” Respondents to surveys show varying levels of agreement with the dimensions measured on surveys, but most people do not reference antipathy toward Jews directly in their day-to-day speech or online. Just as respondents in surveys frequently conceal their real views due to social desirability bias, participants on social media platforms may blunt their views through the deployment of conspiracy narratives.

As Mulhall notes: “Both mainstream platforms, such as Facebook, and largely unmoderated forums, such as 4chan/pol/ and Telegram, are awash with antisemitic conspiracy theories tied to the common thesis such as supposed Jewish influence over governments and world politics (often referred to as ‘ZOG’ for “Zionist Occupied Government” and related ideas, such as the ‘Deep State’)” [9]. Rather than express outright hatred or intended violence toward Jews, a criminal offense in some jurisdictions, the conspiracy narratives on these less moderated forums mostly express common antisemitic attitudes in indirect ways. For example, conspiracy narratives reflect the “hidden power” dimension of antisemitism, casting Jews as puppet masters who infiltrate political groups or wield secret control over government, or they may highlight dual loyalty, namely the notion that Jews’ hidden influence is being exercised for particularistic or even Israeli political ends.

Jikeli et al. [6] found that between January 2019 and August 2020, peaks in conversations about Jews and Israel corresponded to coverage of specific events in traditional media. While the media reports may not in themselves have contained antisemitic content, 11.2% of their sample of tweets containing the word “Jew” were antisemitic. They find that antisemitic content in tweets with the word “Jew” reflected stereotypes related both to conspiracy theories of hidden Jewish power and to stereotypes of distasteful Jewish traits and behaviors. This research demonstrates the link between selected key terms and antisemitic conspiracy narrative content. It also supports the observation that the presence of a particular lexical term does not necessarily correspond to the presence of hate speech or even offensive content [45].

In short, compared to overtly antisemitic attitudes and language, antisemitic conspiracy narratives are less easily recognized as hate and also pose greater challenges for platform detection and moderation, even as they relate strongly to traditionally measured dimensions of antisemitism. Moreover, key explicit terms like the word “Jew” may be used to identify content likely to contain these narratives, but the presence of these terms is not sufficient in and of itself to identify posts as antisemitic. A way to approach to this puzzle, one to which we now turn, is to think of on-line hate as a language game.

### The language game

In practice explicit antisemitic utterances are not costless—these costs may range from social ostracism to deplatforming—and so they are frequently expressed in veiled or implicit ways. Detecting anti-Jewish hate in general and antisemitic conspiracy narratives in particular becomes even more difficult when such narratives may be communicated to an in-the-know audience without explicit mention of Jews. In these cases, users leverage implicit language or “dog whistles” to signal the audience that the conspiracy narratives they are sharing are not about some “nebulous outgroup” [46] but about the Jews.

Tuters and Hagens [46] describe the case of the triple parenthesis meme on 4chan as an implicit signifier of antisemitic messaging:

While one can still find instances in which anons use triple parentheses as an explicitly anti-Semitic slur, notably with terms like ‘jews’, ‘soros’ and ‘kushner’, its dominant nebulous use on/pol/ is abstracted from its original name-calling, to the extent that its anti-Semitic history may even be unknown to those unfamiliar with 4chan’s language games. However, one should assume that the triple parentheses’ ‘real’ anti-Semitic message is clear to those initiated within 4chan’s vernacular subculture, for whom the meme does not appear as a floating signifier at all. Despite this and ongoing appearances of the meme’s original use as an anti-Semitic targeting technique, what also comes to the fore is a type of nebulous use allowing the meme to at once seemingly abstract its referent into a floating signifier while at the same paradoxically reinscribing the narrative of anti-Semitic conspiracy theory into a playful language game – a combination which makes the meme all the more ominous. [p. 2231]

Generalizing from the case of memes to that of implicit language, “language games” allow for construction of an online ingroup “us,” where specific linguistic knowledge is used to demonstrate and negotiate ingroup belonging [46]. At the same time, this ingroup language may be exported to other contexts as “floating signifiers,” in themselves empty of meaning and ready to absorb the meaning imparted by new groups [47]. In this way, such floating signifiers, with their original meaning abstracted, readily become a means for identifying a nebulous other that may appeal to multiple different groups. Yet the ingroup will recognize the original intentions of the terms, while the floating signifier allows for wider dissemination and the possibility of reinscribing the original meaning for a new and previously uninitiated audience.

Consider, for example, the use of Jewish financier George Soros as a key actor in antisemitic conspiracy narratives. In their study of “platformed antisemitism,” Riedl, Joseff, and Soorholtz, et al. [3] find inclusion of the term “soros” in a tweet to be the highest predictor of a tweet being antisemitic followed closely by “rothschild,” with over 80% of tweets containing either name being coded as antisemitic [3]. Yet, the name “Soros” on its own may be a floating signifier. For example, so frequently are the terms “Soros-backed” and “Soros-financed” used as descriptive epithets for liberal politicians and district attorneys on Fox News that the network’s website has devoted an entire page with dozens of links to “Soros” stories [48]. In antisemitic conspiracy narratives, the billionaire George Soros is the modern-day equivalent to the famous Jewish banker Mayer Amschel Rothschild, and references to both men and their families are coded terms for “Jews with hidden power.” At the same time, as a floating signifier, Soros is also used for nebulous othering of liberals or wealthy elites. Indeed, in some contexts the mention of Soros and the use of Soros-backed as an adjective has no intentional relationship with antisemitism; he just happens to be Jewish.

Context, in this case community, matters. As Quaranto [49] notes, “focusing on communities involves considering utterances not in isolation, nor merely in their immediate context, but as performances of pre-existing practices, as acts embedded both in a history of usage and in a social and political history” [37, p. 330]. In a community that plays this particular language game, we therefore argue, the relationship between the implicit term “Soros,” conspiracy narratives, and explicit reference to Jews will have been established in online conversation: Soros would have been explicitly presented as a key antisemitic conspiracy villain or a signifier of hidden Jewish power more generally. When this duly inducted ingroup encounters mentions of Soros in other contexts, the antisemitic message, intended or not, comes through loud and clear. Moreover, the antisemitic connections established by the ingroup are easily discoverable for new audiences that may also be inclined to reidentify Soros as a Jewish villain and direct their negative feelings about the conspiracy narratives in which he stars toward Jews.

We posit that antisemitism is communicated not merely through the expression of a latent structure of attitudes in plain language but through a complex back and forth of explicit and implicit signifiers that relate to antisemitic conspiracy narratives. Moreover, the language game is community dependent, with the clearest expressions of antisemitism taking place in communities with an identifiable ingroup that defines itself against the Jews as an outgroup. To explore the communication of various dimensions of antisemitism through explicit language, implicit language, and conspiracy narratives in an online community, we use the case of QAnon discussion on a mainstream platform, Reddit.

### Antisemitic content on a mainstream platform: expectations and hypotheses

On a mainstream platform with content moderation such as Reddit, we would expect to see a limited amount of explicit content and a larger amount of implicit content due to the potential for social ostracism and/or deplatforming. Given the higher cost of overt or explicit speech relative to implicit speech, we hypothesize:

***H1. Implicit references occur far more frequently in posts than do explicit ones.***
***H2. A larger percentage of users use implicit language compared to explicit language.***

In order for the language game to connect explicit language to implicit language, both would need to appear in the same post. Moreover, to the extent such co-occurrence is intentional, posts with explicit terms would more often include implicit terms than other types of terms. For example, we would expect to see the explicit term “Jew” co-occur more frequently with the implicit term “Soros” than with a term like “politics,” which is neither explicit nor implicit. We hypothesize:

***H3: There is greater co-occurrence of implicit terms with explicit terms relative to other types of terms.***

Moreover, explicit and implicit language would need not only to co-occur but to co-occur in ways that establish the underlying meaning of implicit terms. As indicators of antisemitic signals for the ingroup, implicit terms would need to appear in connection with content that communicates antisemitic attitudes or tropes—dimensions of antisemitism:

***H4: Implicit terms are used in posts containing explicit terms to reflect any of the four dimensions of antisemitism.***

Additionally, these patterns of co-occurrence between implicit and explicit references would point the ingroup to the role of Jews as key conspirators in common conspiracy narratives, such that Jews could also be recognized in generalized versions of the same narrative that do not directly reference Jews. Thus, we hypothesize:

***H5. Posts with explicit content link implicit and explicit reference to Jews and point to the overt role of Jews in common conspiracy narratives.***

Examining the language game requires studying a community engaged with antisemitic content. We focus on subreddits related to QAnon because QAnon has been widely recognized as an antisemitic movement.

QAnon traces its origins to 4Chan’s Politically Incorrect board (/pol/), where on October 28, 2017, an anonymous user claiming (non-existent) “Q level” security clearance posted a thread titled “Calm before the Storm” [50]. Discussions of similar “Q drops” and the far-sweeping conspiracy to which they alluded soon migrated from fringe platforms to mainstream platforms like Reddit, Twitter, and YouTube [50]. The Anti-Defamation League describes QAnon as “a decentralized, far-right political movement rooted in a baseless conspiracy theory that the world is controlled by the ‘Deep State,’ a cabal of Satan-worshipping pedophiles, and that former President Donald Trump is the only person who can defeat it” [51]. Indeed, QAnon brings together multiple conspiracy theories at once to create what Papasavva et al. call a “super-conspiracy theory” [52]. Many of the visceral conspiracy narratives upon which QAnon draws are historically antisemitic [53]. Key among them is a narrative of blood libel—the Jews stealing the blood of Christian children—that dates back to the middle ages as well as the “Deep State” narrative, wherein the elites and globalists in the controlling cabal are Jews or are working on behalf of a “Zionist-occupied government” (ZOG) and seeking to assert a “New World Order” (NWO) to enslave humanity [51,53,54]. While these narratives contain traditional and well-recognized antisemitic tropes, they also have generalized versions focused on an elite cabal, the Deep State, and the New World Order. In these more generalized versions, the conspiracy narratives are stripped of the mention of Jews or ZOG. Yet in the QAnon subreddits, those in the ingroup would be initiated into the language game wherein these narrative references have antisemitic undertones, although they are not explicitly antisemitic.

## Methods

### Data and sample

We use a body of posts from two QAnon subreddits from their creation to their deplatforming. The data were scraped from the Push-Shift API and were gathered from activity on the subreddits r/greatawakening and r/CBTS_Stream, both of which were deplatformed due to threats of violence, r/CBTS_Stream in March 2018 and r/greatawakening in September 2018. Our use of the data comply with Reddit’s terms of use. The Queens College-CUNY IRB approved the study protocol and designated it as an “exempt” category of human subjects research.

**Fig 1:**
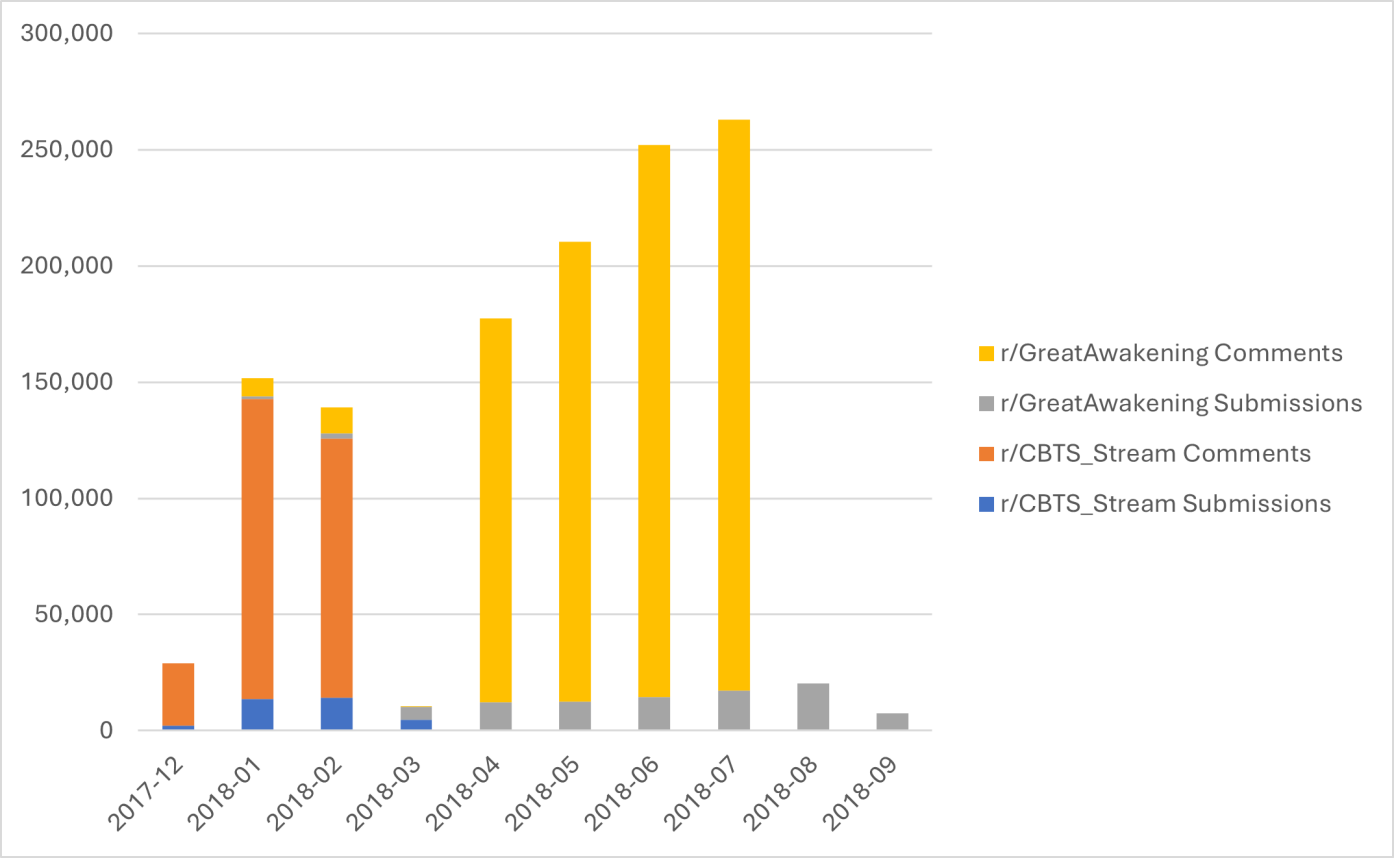
QAnon Subreddit Submissions and Comments.

Reddit activity (collectively, *posts*) takes one of two forms: a *submission* is the initial post in a thread, and *comments* are responses posted within the thread. Comments may be a reply directly to a submission or may be connected to another comment within the submission’s thread. In addition to the text of the post, the corpus includes date/time information for both submissions and comments, the thread title (submissions only), author information (when available), and sufficient information for connecting comments to the thread and/or other comment they are referencing. Our corpus consists of over 1.26 million posts (see Fig 1) to these subreddits between December 2017 and September 2018, including all submissions for both subreddits (*n* = 128,269) and comments from all months, with the exception of March 2018 (1,132,998). The comment data for March 2018 was not available from PushShift. While this is unfortunate, the gap is not large enough to change the conclusions of this study.

### Measures

To identify explicit and implicit antisemitic speech, we use publicly available and expert generated lists of keywords to filter the corpus. The selected terms have been associated with antisemitic discourse, although it should be noted that their presence in posts does not always indicate antisemitic content.

### Explicit antisemitic speech

We use the hate speech dictionary developed by Siegel, Nikitin, Barberá et al.[35], which builds on a number of hate speech databases, including: Hatebase and the Racial Slur Database, comprehensive online repositories of global hate speech [36,37], and the Anti-Defamation League’s database of slogans, terms, and symbols used by white-nationalist groups [55]. Hate speech labels in this dictionary include Anti-Asian, Anti-Black, Anti-Immigrant, Anti-Latino, Anti-Muslim/Anti-Arab, Anti-Semitic, Homophobic/Anti-LGBTQ + , Misogynistic, and White Nationalist. We focus exclusively on the antisemitic terms.

We have further augmented this dictionary by including singular and plural instances of the various terms when these were not initially included.

We define explicit language as language labeled “Anti-Semitic” in the hate-speech dictionary or as terms that have been associated with antisemitic hate speech but that are not included in the hate-speech dictionary. These additional terms were part of an expert-generated list (described below) and include direct references to Jews, Judaism, or Zionism, for example, “Jewish,” and “Hebrew.” It also includes terms that could be considered antisemitic hate speech but that are not included in the Hate-Speech Dictionary, for example, words with alternative spelling to those on the original list. The dictionaries and data used in this paper are available at https://github.com/e2unlimitedtech/QAnonSubreddits.

### Implicit antisemitic speech

Two of our co-authors, Jeffrey Kopstein and David Frey, have extensive scholarly expertise related to antisemitism and the Holocaust. They collaborated to assemble a list of terms strongly associated with antisemitic tropes and antisemitic conspiracy theories. Some of these terms (as described above) were explicit references to Jews, but the majority were implicit terms—terms often prominent in antisemitic conspiracy narratives but that do not explicitly reference Jews. This list includes terms and names that have historically been used as indirect references to Jews. These include the names of influential Jews (or people thought to be Jewish), for example, Soros, Bloomberg, and Rothschild (including a variety of common misspellings). Indirect references also include terms like “banker,” “elite,” “globalist,” and “cosmopolitan,” as well as common antisemitic conspiracy phrases like “New World Order,” “Deep State,” and “Great Replacement.” The expert-generated list also includes Holocaust references, a specialized type of indirect reference that includes, for example, the names of concentration camps, references to Nazis, and Holocaust denial terms like “holohoax.” Finally, the list includes reference to Israel or Israeli politics, for example, “Israeli” and “Palestinian.” Thus, our expert-generated list contains implicit references to Jews and Israel that may or may not be signals of antisemitic discourse. Each term in the expert generated list was labeled as explicit or implicit, and all antisemitic terms from the hate-speech dictionary were included as explicit terms.

### Quality checks, exclusions, and term aggregation

The hate-speech dictionary and expert-generated antisemitic terms lists both contain a number of context-specific terms, some of which were present on social media but not used in the way specified by the dictionaries. For example, “bar code,” was identified in the hate-speech dictionary as a reference to the tattoos given Jewish prisoners in some concentration camps, but in the sample posts, it invariably related to more common uses of the term, specifically to actual bar codes. Two coders validated the terms on both lists by taking a random sample of five posts that included the term (when available, otherwise a smaller set) and coding for usage consistent with inclusion in the dictionaries. Inter-rater reliability was over 99.8%. We exclude from the dictionaries terms for which fewer than half the cases represented dictionary-consistent usage. In total, we include 892 explicit terms and 278 implicit terms in our list for initial detection.

Before processing, each post was converted to lowercase and non-alphanumeric characters were removed. To improve the interpretability of our results, we combined terms that refer to the same entity into a single (“roll-up”) umbrella term. This approach is borrowed from Tangherlini et al., who refer to their umbrella groupings as “supernodes.” For instance, the philanthropist George Soros is referred to as both “Soros” and “billionaire George Soros” throughout the corpus of posts; after preprocessing, both terms were replaced with the designator “_SOROS_,” with the underscore and capitalization used to denote supernodes. In order to find such co-referring mentions of entities, we use network graphs of the most frequently co-occurring terms and manually select those which refer to the same entity. After cleaning posts and combining relevant entities into a single designator, the remaining terms were lemmatized, thus reducing a word to its root (e.g., rocks, rocking, and rock will all be representing as “rock” in the data), using the standard WordNet Lemmatizer as implemented in the Natural Language Toolkit (NLTK) package in Python. A standard English language stopword list from the NLTK package was also applied to the text, to remove frequently occurring words that often to do not contribute to text analysis (e.g., “and, the, how,” etc.). Additionally, consistently with common practice in text analysis, terms with fewer than 3 characters, that did not appear in a minimum of 10 posts, or that appeared in more than 30% of the posts in the corpus were removed. The terms that remained after the cleansing process were formed into tokens, with groupings of single terms (unigrams), two terms (bigrams), and three terms (trigrams) considered.

### Dimensions of antisemitism and conspiracy narratives

For each implicit term in our dataset, we took a random sample of three sentences where the term appears with explicit terms. A team of coders determined whether the terms related to any one of the four dimensions of antisemitism: hidden Jewish power, dual loyalty, Holocaust minimization or obfuscation, and undesirable Jewish traits or behaviors. These coded data are available at https://github.com/e2unlimitedtech/QAnonSubreddits. Interrater reliability identifying dimensions averaged 82.3%, ranging from 69.9% to 95.1% per dimension. We also coded the same examples for whether they include reference to specific antisemitic conspiracy narratives (described in the Results section). Inter-rater reliability for association of particular narratives to sentences averaged 94.4%, with a min of 87.4% and a max of 99.6%. Finally, we coded for whether the implicit term in the sentence referenced Jews or a uniquely Jewish trait, using the conservative criteria that the reference be clear from the sentence itself and not those preceding or following in the larger post. Inter-rater reliability for determining the references is 86.6%. Of the 82 implicit terms and supernodes found in the corpus, 95.1% appeared in sample sentences that invoked a dimension of antisemitism, related to an antisemitic conspiracy narrative in our set, or directly referenced Jews or uniquely Jewish traits. The exceptions, which showed no such associations in our sample posts, are: “_BDS_,” “_BIRKENAU_,” “_BENJAMIN_,” and “_WORLDGOVERNMENT_.”

## Analysis

### Implicit and explicit word frequency

To test ***H1: Implicit references occur far more frequently in posts than do explicit ones***, we examine word frequency by post, comparing the frequency of both implicit and explicit antisemitic terms in submissions and comments to determine the relative frequency. We also examine the most common explicit and implicit antisemitic terms. We further analyze the extent to which users deployed explicit or implicit antisemitic language in their posts to determine the support for ***H2: More users will use implicit language versus explicit language***.

### Implicit and explicit term co-occurrences

To examine evidence of a language game, in support of ***H3: There is greater co-occurrence of implicit terms with explicit terms relative to other types of terms***, connecting explicit references to implicit ones, we examine the co-occurrence of terms from our explicit and implicit terms lists across comments and submissions. We examine the co-occurrence of explicit language with terms from our implicit list compared to other terms in the corpus. We use a chi-square test to determine whether there is a significant difference in co-occurrence for implicit terms compared to others.

To test ***H4: Implicit terms are used in posts containing explicit terms to reflect any of the four dimensions of antisemitism*,** we examine the percentage of implicit terms appearing in the corpus in ways that reflect each of the four dimensions of antisemitism.

Finally, to test ***H5. Posts with explicit content link implicit and explicit reference to Jews and point to the overt role of Jews in common conspiracy narratives,*** we generated an undirected network that shows the patterns of co-occurrence for implicit and explicit terms. The graph shows nodes which have edges with co-occurrence frequency (edge weights) of 100 and above. We do not limit inclusion based on degree centrality, but we only kept terms that were in our rollup list (controlled vocabulary) and had a minimum document frequency of 300 and a maximum document frequency of less than 30% of the total number of documents in the corpus.

The network graphs allow for an examination of the relationship between explicit and implicit terms, examining both the co-occurrence of these different types of terms with each other as well as the community of content to which various terms contribute. Furthermore, we analyze a sample of 246 randomly selected sentences containing both implicit and explicit terms, three for each of the 82 implicit terms in the data. We examine the percentage of implicit terms associated with posts containing references to specific antisemitic conspiracy narratives, which would establish their status as floating signifiers of these narratives when they appear on their own.

## Results

Is explicit antisemitic language more costly to use than implicit language, such that implicit language appears more frequently in online conversation (***H1***) and a broader range of users are likely to use implicit language than explicit language (***H2***)? We first analyze the document frequency of implicit and explicit language both by type of document and by individual users.

### Implicit and explicit word frequency

Implicit antisemitic terms appeared more frequently in the subreddits than did explicit antisemitic terms. Explicit antisemitic language appeared in 0.66% of the posts overall, while implicit terms appeared in 8.6% of the posts. Submissions contained similar proportions of explicit antisemitic terms compared to comments, 0.56% compared to 0.67%, but contained higher proportions of implicit antisemitic terms than comments, 12.76% compared to 8.13%.

**Fig 2:**
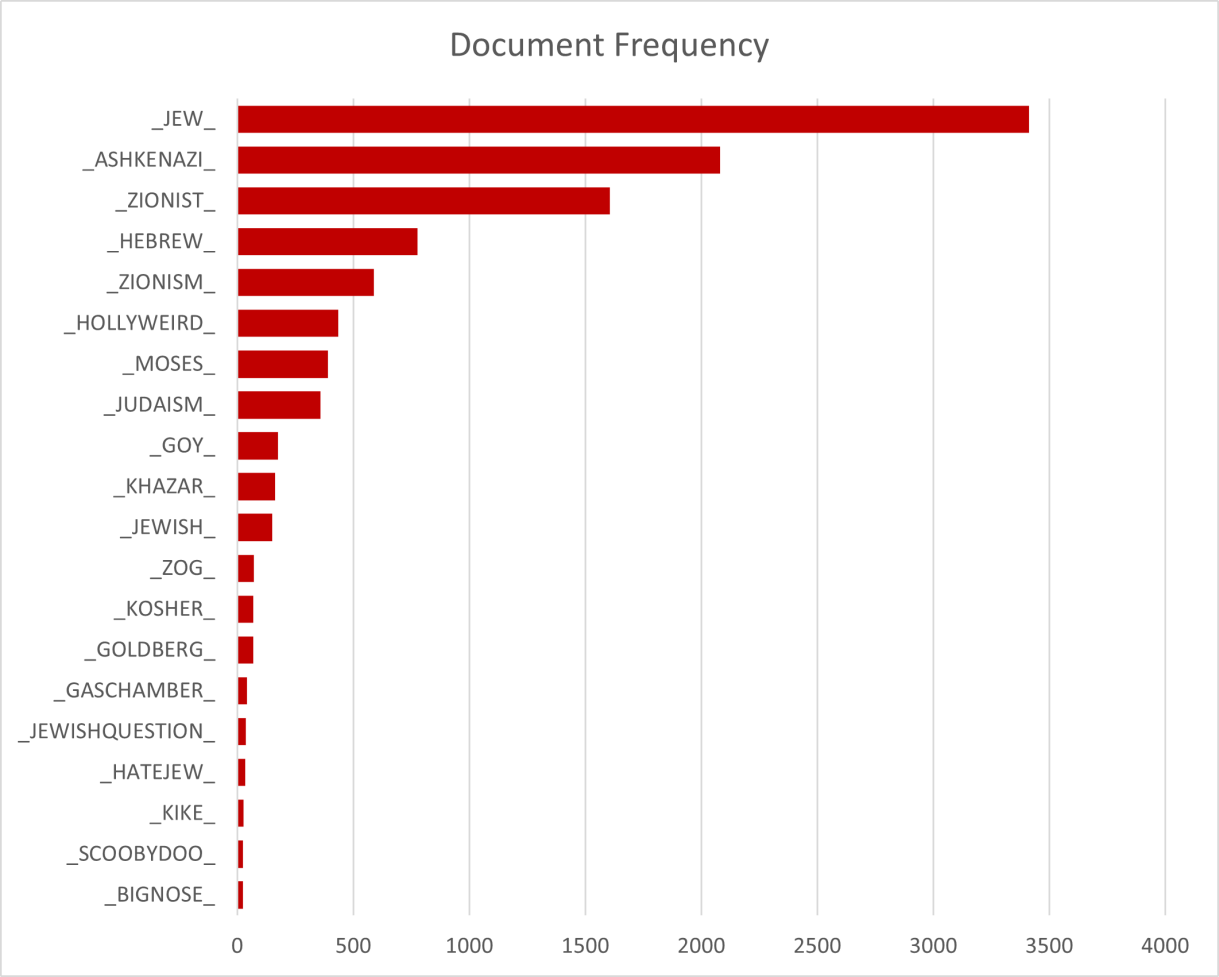
Document Frequency of Top 20 Explicit Antisemitic Terms.

The most common explicit antisemitic terms in the list point to Jews or groups and ideologies directly related to Jews, such as Zionists and Judaism. Ethnic slurs, though appearing among the most frequently used explicit terms, were relatively rare. Among the 20 most frequently used explicit terms, the minimum post frequency was 24 and the maximum post frequency was 3,414 documents. For explicit terms, the terms with the highest quantity of appearances were: _JEW_, _ASHKENAZI_, _ZIONIST_, _HEBREW_, and _ZIONISM (Fig 2).

**Fig 3:**
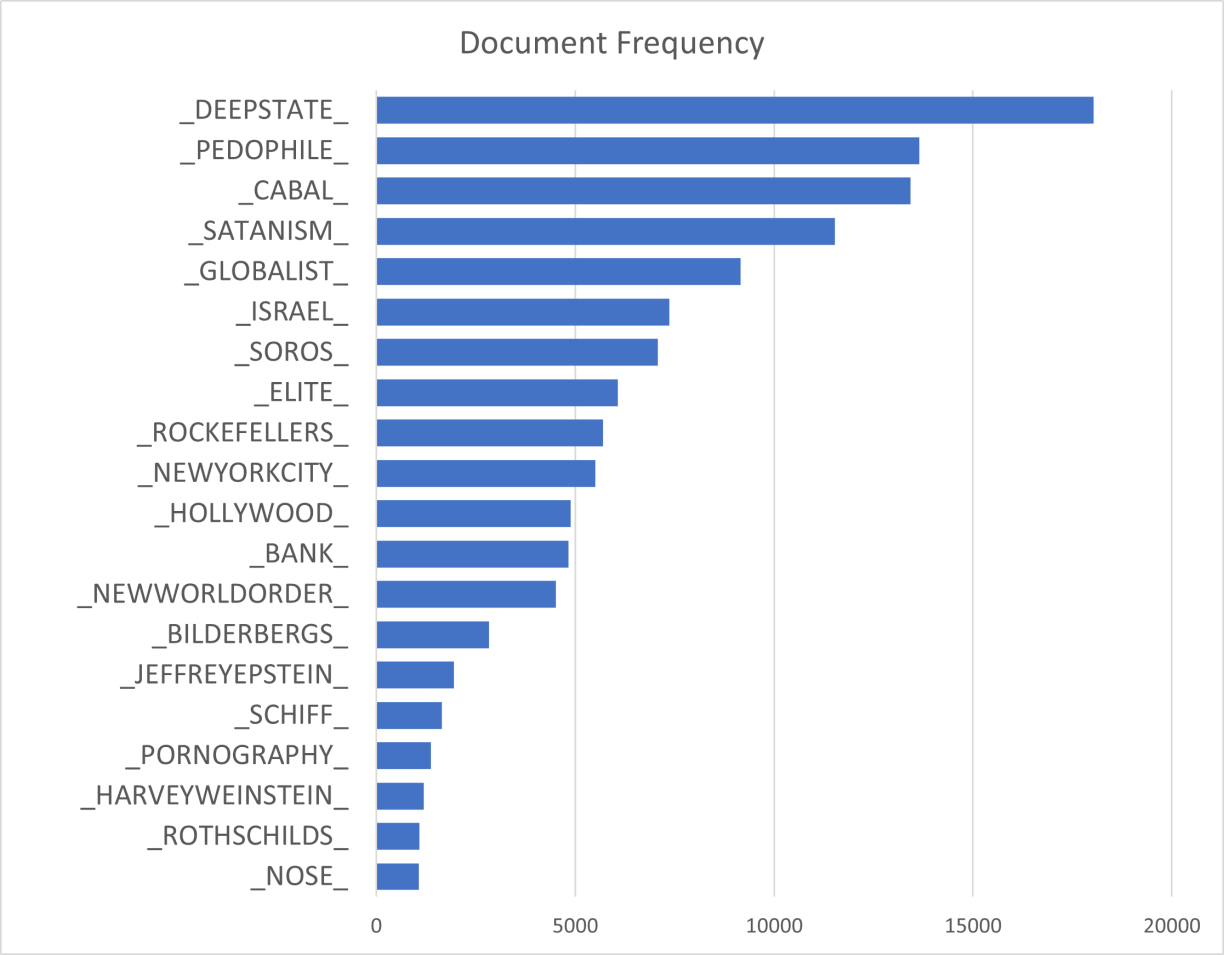
Document Frequency of Top 20 Implicit Antisemitic Terms.

The 20 most used implicit terms appeared far more frequently (Fig 3), ranging from a minimum of 1,072 documents, in this case posts, to a maximum of 18,035. The top implicit term, _DEEPSTATE_, appeared in 18,035 posts, far more than any other term. In contrast, the next most frequently deployed terms showed varying but relatively high frequencies: _PEDOPHILE_, _CABAL_, _SATANISM_, AND _GLOBALIST_.

### User behavior

Of the 34,500 users (excluding deleted and AutoModerator users) who posted at least once to either subreddit, only 6.84% authored submissions or comments with explicit antisemitic content (most of these also included implicit antisemitic content as well). In contrast, 27.95% posted submissions or comments containing implicit antisemitic terms, but not explicit terms; thus 34.79% of authors on these subreddits engaged in plausibly antisemitic content generation. This suggests that more than a third of QAnon users in our sample shared antisemitic conspiracy narratives, knowingly or unknowingly.

Thus, we find support for ***H1: Implicit references occur far more frequently in posts than do explicit ones***, on a moderated, mainstream platform than is explicit language. Moreover, we find support for ***H2: More users will use implicit language versus explicit language***.

### Implicit and explicit term co-occurrences

We next turn to the hypothesized language games connecting implicit and explicit language. Does the occurrence of explicit and implicit language encode implicit language and the conspiracy narratives it references as antisemitic? We consider the patterns of co-occurrence of implicit and explicit terms compared to others to test whether implicit terms have stronger association with explicit terms than do other terms in the corpus (***H3***). We then examine the nature of this association, to determine if it is random or whether it points to dimensions of antisemitism (***H4)***, and to the role of Jews in common conspiracy narratives (***H5***).

Despite their relative infrequency across submissions and comments, posts with explicit antisemitic terms show strong patterns of co-occurrence with implicit antisemitic terms. Table 1 shows the cross-tabulation of implicit and explicit terms for all posts (submissions and comments).

**Table 1:** Frequency of implicit/explicit language in the QAnon corpus.

	Number of posts that contain implicit terms	Number of posts that do not contain implicit terms	Row totals
Number of posts that contains explicit terms	4,466	3,860	8,326
Number of posts that do not contain explicit terms	103,756	1,149,185	1,252,941
Column Totals	108,222	1,153,045	1,261,267

For the null hypothesis, “Explicit language appears with the same frequency with implicit language as with other terms,” the chi-square statistic is 21,693.22 and the p-value is < 0.00001. We thus reject the null hypothesis in favor of the alternative hypothesis, ***H3: There is greater co-occurrence of implicit terms with explicit terms relative to other types of terms***.

## Dimensions of antisemitism

Do the co-occurrences of explicit and implicit language establish content as antisemitic by communicating dimensions of antisemitism?

**Fig 4:**
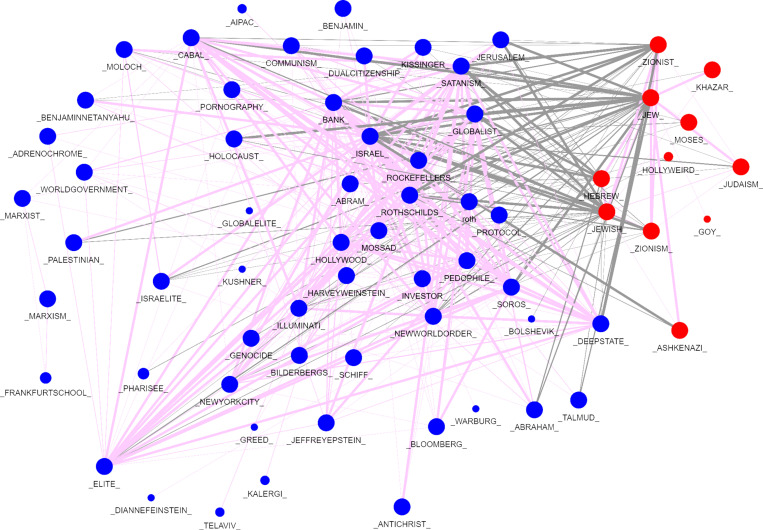
Network Graph of Connections Between Explicit and Implicit Terms.

Fig 4 shows a network graph of the co-occurrences of both the explicit and implicit terms in our corpus. The connections between the explicit terms (in red) on the right of the network graph are shown with the implicit terms (in blue) on the left. In this web of connections, the most common explicit terms are not antisemitic hate slurs but rather direct references to Jews or terms associated with Jews.

Using the entire corpus, the graph in Fig 4 shows that the implicit terms most strongly connected to explicit terms refer to hidden powers working behind the scenes (for example, bank, cabal, globalist, elite, world government, deep state, Illuminati, and names of powerful people or families) and to sacrifice or mistreatment of children (for example, Satanism, Moloch, pedophile, pornography, adrenochrome).

A further examination of posts with these co-occurrences illustrates that these linkages are deliberate rather than happenstance. For example, a user provides the map between explicit (in red font) and implicit (in blue font) references, letting other users know that the banking families, banking dynasties, and the global cabal are indeed the Jews:

I suspect the [13 **Jew** *banking* dynasties](https://www.disclose.tv/the-13-illuminati-families-who-secretly-rule-the-world-313144) who basically own the world and [start the wars](https://i.imgur.com/rGB41vE.jpg) and who [own our Associated Press} (https://i.imgur.com/dcXQT98.jpg) are the *cabal* who fund and orchestrate the entire *globalist* attack against America and western civilization. My theory is these **Jew** *banking* families usually use their power and influence to ensure either nominee are just *globalist* shills wearing either a Democrat or Republican mask…. I suspect our **Zionist** overlords usually allow us to hold our meaningless elections because it makes no difference to them when they control both sides of the uni-party. This is a far simpler solution than attempting to script an entire election as a theater production. I suspect the only reason the **Zionist** plan was derailed in 2016 was because they underestimated just how much patriotic Americans hated our diversity hire homosexual Kenyan Muslim President and how much they loathed Hillary Clinton.

Not only does the post identify various groups as being Jewish, but it also insinuates that politicians and the media are controlled by Jews.

The patterns of connections in the above quoted post and in the network graph among the explicit and implicit terms in our dictionaries establish the connectedness of these terms. Thus, for example, while terms referring to banks and bankers, rolled up into the term “_BANK_,” might not at face value seem to be referring to Jews, the pattern of co-occurrences between explicit and implicit terms establishes that for the QAnon ingroup references to banks in the language game are veiled references to Jews or to notions of hidden Jewish power, and these connections are developed in a small percentage of posts with explicit language. Similarly, another post draws the connections between implicit and explicit mention of Jews:

I am old enough to remember when our source of TV news was NBC ABC and CBS. It was almost all cookie-cutter (thanks CIA) news. We were being lied to back then: misinformation and mistruth were the two pillars. Public opinion was easy back then to manipulate. For those like H.G. Wells mass communication such as the television meant new paths for social control far beyond anything in the past. And yes it was working until the Internet and the smart phone came along in which everyone was potentially a reporter/investigator. The old cookie-cutter media was having a problem. The problem was that its lies were being exposed. As strange as it sounds we found the source of those lies it was what Churchill referred to as the High *Cabal*. It began with Mayer Amschel *Rothschild* who gave support to the **Ashkenazi Jew** Adam Weishaupt who founded the *Illuminist* Socialist movement in the Bavarian town of Ingolstadt on the first of May 1776 which subsequently infiltrated and undermined Freemasonry. Sound like a conspiracy? It was and still is. And there are plenty of facts to back up this conspiracy. You can find these facton YouTube or the Internet. It’s all there.”

Here again, a user explicitly connects the notorious cabal, the Rothschilds, and the Illuminati to the Jews in a post that emphasizes hidden Jewish power.

In all, eighty-two implicit terms or supernodes appeared in the corpus. Of these, 85.4% co-occurred in sample sentences with explicit language where the implicit terms related to one or more dimensions of antisemitism. Of these implicit terms, 56% appeared in sample sentences referenced hidden power, 20.7% dual loyalty, 13.4% Holocaust minimization or obfuscation, and 57% distasteful traits; terms could be used to reference more than one dimension. This result from the smaller sample of sentences reflects the patterns in the network graph from the full set of posts with explicit content, namely that the most common dimensions of antisemitism communicated in the QAnon posts reflect notions of hidden Jewish power and undesirable Jewish traits.

In all, we find support for ***H4: Implicit terms are used in posts containing explicit terms to reflect any of the four dimensions of antisemitism***.

### Conspiracy narratives

Do the co-occurrences of explicit and implicit language establish the identity of Jews as villains in conspiracy narratives, such that Jews could be inferred to be the key conspirators even when they are not referenced directly?

**Fig 5:**
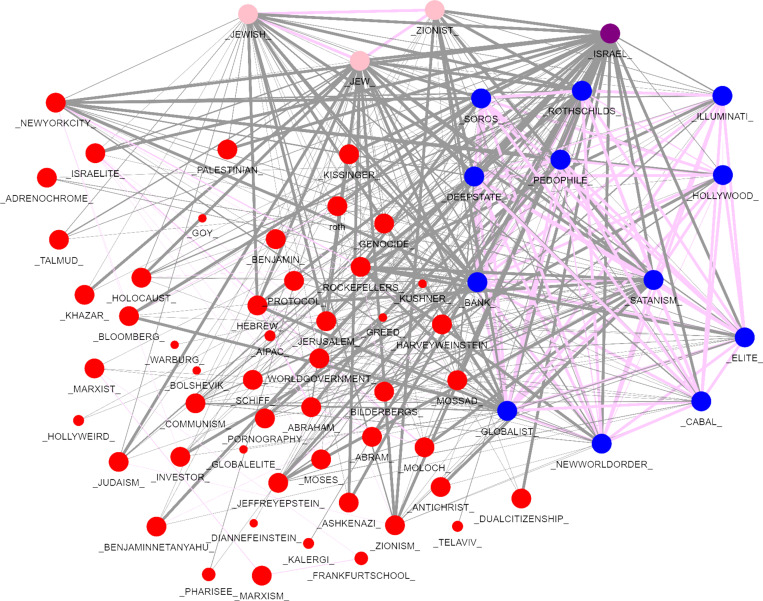
Network Community Clusters of Antisemitic Language.

We examine the way the network of explicit and implicit terms clusters into four content areas or communities as shown in Fig 5.

The network graph in Fig 5 shows both implicit and explicit terms, but this time the colors represent community clusters. Note that “Jew” and “Jewish” and “Zionist”—all frequent explicit terms—are in the pink cluster, and Israel has its own cluster (purple). The blue cluster contains implicit terms related to antisemitic conspiracy narratives of hidden Jewish power (“cabal,” “elite,” “deep state,” “New World Order,” “Soros,” “Hollywood,” “Rothschild”, globalist,” “bank,” “Illuminati”) as well as distasteful Jewish traits associated with blood libel (“pedophile” and “Satanism”). The red cluster connects the more explicit and implicit conversations primarily through names, ideologies, and biblical references. Most of these implicit terms refer to entities or actors, making them potential protagonists in a narrative.

Examining the content of posts with terms from these four clusters, we can once again see the combinations of language (with terms’ font matching the color of their communities from Fig 4), this time designed to convey antisemitic conspiracy narratives. For example, as one user explains:

President Trump is spearheading a global movement against the communist rule of the billionaire **Jew** families (((*Rothschilds*))) (((*Soros*))) (((**Rockefeller**))) (((**Bloomberg**))) (((Cohen))) (((Du Pont))) (((Koch))) and (((Vanderbilts))). He is leading a nationalistic movement to save western civilization. I’m hoping that we’ll see President Trump strip these **Jew** *elitists* of their cash and power and start a revolution against their *banks* by ending the fed (some time in his second term).

This post links the Jews explicitly to global conspiracy narratives related to communism, banks, and to the names of alleged Jewish perpetrators. The three parentheses surrounding the names further signals that these are considered (sometimes erroneously) to be Jewish names [46]. In another example, a post connects a long list of presumably Jewish names to global conspiracies:

I’m seeing a trend too a certain commonality between people like (((**Adam Schiff**))) and the most powerful members of the Democrat Party including including (((**Chuck Schumer**))) (((**Adam Schiff**))) (((Bernie Sanders))) (((Richard Blumenthal))) (((Eliot Engel))) (((Nita Lowey))) (((Steve Cohen))) (((Jerry Nadler))) (((Sender Levin))) (((Brian Schatz))) (((Jared Polis))) (((Brad Schneider))) (((Alan Lowenthal))) (((Ben Cardin))) (((Jan Schakowsky))) (((David Cicilline))) (((Jacky Rosen))) (((Jamie Raskin))) (((Lois Frankel))) (((Ted Deutch))) (((Brad Sherman))) (((Susan Davis))) (((John Yarmuth))) (((Ron Wyden))) (((Michael Bennet))) and (((Josh Gottheimer)))? I’ve noticed a similar commonality between all of the most powerful *globalist* families who are trying to destroy President Trump and Western civilization... such as the (((*Rothschilds*))) (((*Soros*))) (((*Rockefeller*))) (((*Bloomberg*))) (((Cohen))) (((Du Pont))) (((Koch))) and (((Vanderbilts)))......and our most liberal *globalist* Constitution-hating Supreme Court Judges such as (((Ruth Ginsburg))) (((Elena Kagan))) (((and Stephan Breyer))) have something in common too... [...and (((*George Soros*))) funds all of the worst Republican swamp-monster RINOs?](http://www.breitbart.com/big-government/2017/02/06/records-soros-fund-execs-funded-paul-ryan-marco-rubio-jeb-bush-john-mccain-john-kasich-lindsey-graham-in-2016/) [...supported by the fake news...] (https://img.4plebs.org/boards/pol/image/1503/34/1503340078185.jpg) [...including 80% of President Trump s Fake News Award winners...] (https://imgoat.com/uploads/8dd2c7955c/76964.jpg) Weird huh?

In this example, Trump, along with the whole of western civilization, is the intended victim of conspiracies by globalist families and of public officials all identified as Jewish.

Finally, we examine a sample of 246 sentences, three randomly selected sentences for each of the 82 implicit terms detected in the data that contain both the implicit term and explicit terms. Of the 82 implicit terms, 82.4% were used to refer to Jews or to uniquely Jewish traits. Additionally, 82.9% of the implicit terms were used in sentences with explicit language that enumerated one or more antisemitic conspiracy narratives.

**Fig 6:**
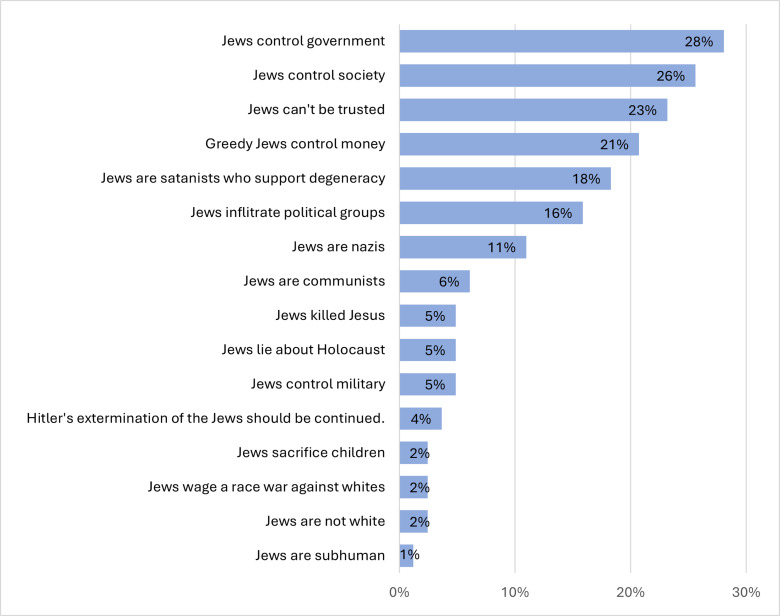
Frequency of Narratives Related to Implicit Terms (N = 246 sentences).

Fig 6 displays the specific narratives for which we coded and the percentage of terms from our sample of examples that correspond to each.

The central conspiracy narratives in QAnon relate to the power-wielding of an elite cabal that controls government, society, money, and political groups by Satanists who support degeneracy, including child abuse. The combination of these narratives with explicit mention of Jews alters these QAnon narratives from general conspiracy narratives to specifically antisemitic ones: the Jews are the cabal that controls the government and society against the interests of the American people, and they are the dehumanized villains involved in satanic blood rituals and other abuse of children. Thus, given this roadmap contained in posts with explicit language, QAnon users can recognize the language game when they encounter conspiracy narratives in other posts that do not mention Jews or Zionists. For the purposes of the ingroup’s language game, these posts containing explicit and implicit language together announce that conspiracies involving the cabal, the elite, the banks, and the Satanists, for example, all involve the Jews or Zionists, even when these conspirators are not directly named.

We therefore find support from our analysis of networks, the examples showing term mapping, and our analysis of the co-occurrence of implicit and explicit terms in posts referencing specific antisemitic conspiracy narratives for ***H5. Posts with explicit content link implicit and explicit reference to Jews and point to the overt role of Jews in common conspiracy narratives.***

## Discussion

Studies of antisemitic content on social media report that users present implicit language and conspiracy narratives rather than explicit language to communicate antisemitism in ways that evade platform detection and social stigma. In this paper, we formally describe and then test the relationships between explicit language, implicit language, and conspiracy narratives. We show how an antisemitic community on a mainstream platform establishes for its ingroup the meaning of implicit terms and the intended reference to Jews as villains in more generalized conspiracy narratives. We have provided a generalized method for examining how hate is subtly expressed in online communities. The findings have implications for tracking the fast-moving changes in encoded language use in communities, not only in relation to antisemitism but also to other group-based forms of hate.

Using both content network graphs and qualitative coding, we find observable patterns of co-occurrence of implicit and explicit terms that point to various “reasons” to dislike Jews, the dimensions of antisemitism. These patterns of co-occurrence also establish implicit terms as references to Jews, and they communicate conspiracy narratives with a direct naming of Jews as the conspirators. Given the relatively rare occurrence of explicit language in the QAnon subreddits, we find that such content accounts for a very small portion of the corpus. Yet this combination of explicit and implicit content serves a crucial ingroup communication function in the language game, providing a linguistic entry portal to ingroup users while defining the ingroup in opposition to a hated and dangerous other.

At the post and even at the sentence level, these co-occurrences operate to provide the ingroup with a roadmap or dictionary for interpreting the meaning of implicit terms and generalized conspiracy narratives when they occur without direct reference to Jews. Only a small group of users, fewer than 7%, employ explicit language and, in so doing, establish a sort of Rosetta stone for the rest. A broader group, more than a quarter of users, are then able to share only implicit content that the community then knows to interpret as antisemitic content. Yet such content might be difficult for the platform or even for audiences outside of the community to detect or confirm as antisemitic. In all, more than a third of QAnon users actively participated in the language game by employing either explicit or implicit antisemitic language, suggesting that antisemitic content provided a key set of talking points and cultural capital [56] for a broad group of users. Users who did not post such content may have been passive participants in the language game, consuming it by reading this type of content but not contributing to it.

The hate-speech dictionary that we used contained a lot of outdated terms and some that are not necessarily explicit, while the expert-generated list contained a large number of implicit terms that appeared very rarely or not at all in the corpus. Both of these issues point to the importance both of context and of time: the language used by groups may be somewhat unique and also may change over time. There may be explicit and implicit terms that should have been included but were not. Moreover, characterization of certain terms as explicit may have skewed some of the results, although we submit that such potential terms had relative low frequency.

The patterns of co-occurrence between explicit and implicit language, particularly for the most commonly found terms, suggests that a starter set of explicit terms—for example, Jew and Zionist and their variations—might have been used to reveal other commonly used explicit terms as well as implicit terms. This could be done simply by examining the frequency of co-occurrence and the proportion of appearances of terms that included these initial explicit terms and then coding for whether they refer to Jews, pertain to various dimensions of antisemitism, or relate to antisemitic conspiracy narratives. In other words, leveraging the properties of the language game, a very conservative starter list could be used to identify a subset of words and phrases in a corpus and then further investigated to determine whether they are implicit references to Jews and antisemitic attitudes and narratives. Future research will explore this potential methodology and the mathematical qualities of terms within a corpus that would make them good candidates as implicit references or “dog whistles.”

Ultimately, this paper points to the role of language in group dynamics. The language game we observe establishes an ingroup in relation to one or more outgroups through shared language, stories, and meanings. To communicate these shared understandings requires that terms and meanings are defined and made clear somewhere in the group’s discussion, providing something akin to a Rosetta stone or decoder for group members. On a mainstream platform, the relative rarity of content that lays out intended meanings ensures that this knowledge is visible to group members who engage with large swaths of the group’s discussion—to the ingroup.

We now return to the initial question asked in this paper, how can we determine whether content is antisemitic? Our analysis suggests that this determination depends upon the community context in which content is shared more than on any one particular user’s expressions or intentions. If a community is involved in an antisemitic language game, this game may easily be detected, as we have shown, by examining the co-occurrence of explicit and implicit language, particularly in relation to dimensions of antisemitism and antisemitic conspiracy narratives. Posts containing both explicit and implicit language provide the ingroup community with a method for decoding implicit conspiracy narratives. As we have shown, such posts outline the relationship between explicit and implicit terms, in this case identifying common conspiracy narrative villains specifically as Jews. When these conspiracy narratives appear without explicit language, as they more commonly do, ingroup users, who are familiar with the language game, are then enabled to reimpose this specific Jewish identity on implicitly described conspiracy narrative villains. What happens when these terms appear in a different context outside of the community as floating signifiers? When the ingroup encounters these terms in other contexts, they may read into them the meaning that the group has established while outsiders might ascribe different meanings, making these floating signifiers “dog whistles” or terms that carry different meanings for ingroup and outgroup audiences [57–60]. As floating signifiers, these implicit terms are easily disseminated by individuals who intend antisemitic meanings and those who do not. The more pervasive these floating signifiers become, the easier it is at a later time to reinscribe the hate-filled meaning to wider audiences that already subscribe to the generalized conspiracy narratives attached to them. To return to our example of the floating signifier “Soros,” most of the generalized conspiracy narratives about him relate to his wielding of hidden power and influence. At least part of the audience that subscribes to these conspiracy narratives could easily adopt the antisemitic version, wherein Soros is a representative Jew wielding hidden Jewish power, should their own communities begin to identify Soros explicitly as a reference to Jews.

Implicit references and generalized conspiracy narratives provide a vehicle for spreading and engaging antisemitic content with seeming impunity. This may be one important way antisemitism “works” online. These terms and narratives circumvent platform censorship and avoid social stigma and for these reasons are easily introduced and spread to new communities and platforms. Insidiously, they provide an opportunity to leverage common ground around generalized conspiracy narratives with new users without the stigma of explicitly antisemitic content. Yet their intended antisemitic meanings are readily reinscribed for receptive new audiences. In this way, divorcing implicit language and generalized conspiracy narratives from their explicitly antisemitic interpretations enables hate-fueled antisemitic narratives to move from the fringe to the mainstream and to engage a growing audience.

Throughout this paper, we have described conspiracy theory content as subtle in relation to explicit expressions of hate, and certainly such content is more difficult to detect on social media than more overt content. Yet conspiracy theories may also provoke violence [17,52] and are not innocuous despite their greater subtlety [50]. Our findings suggest that curbing the dissemination of conspiracy theory content would likely be helpful in inhibiting the spread of antisemitism online as well as the spread of hate-fueled violence. Of course, curbing speech of any sort online is difficult, and the contribution of this paper lies not in prescription but in diagnosis, highlighting the nature and detection of the problem. This link between conspiracy theories, antisemitism, and violence also suggests avenues for future research about when belief in conspiracy theories promotes violent action and the extent to which violence relates to a community’s explicit identification of the “conspirators.”
